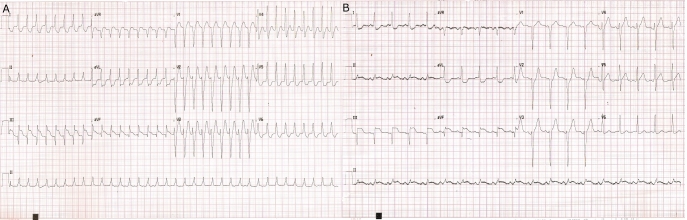# Unusual Presentation of Acute Myocardial Infarction

**DOI:** 10.1016/s0972-6292(16)30547-2

**Published:** 2012-09-01

**Authors:** Anandaraja Subramanian, Aswin Lysander

**Affiliations:** IGGGH & PGI, Pondicherry, India

**Keywords:** supraventricular tachcyardia, acute myocardial infarction

A 49 year-old-man presented to the emergency room with sudden onset palpitation of four hour duration. He also complained of retrosternal chest discomfort and giddiness. There was no history of palpitations in the past. Twelve lead ECG (Figure 1A) showed regular narrow complex tachycardia at the rate of 208/min. No clear P waves were seen. There was ST segment elevation in inferior leads and ST depression in leads I, AVL, V5 and V6. Intravenous 6 mg of adenosine terminated the tachycardia. Sinus rhythm ECG showed ST segment elevation in inferior leads and ST segment depression in lead I and aVL (Figure 1B). Patient received thrombolytic therapy for acute myocardial infarction (AMI) with good result and subsequent hospital course was uneventful. Supraventricular tachycardia, especially at faster rates, usually has ST-T changes (depression) because of repolarization abnormalities or due to P waves falling on T waves. ST segment elevation is uncommon during SVT and myocardial ischemia should be considered in this setting. AMI can have many unusual presentations. Patients may present with abdominal pain, altered mental status, fatigue and weakness. AMI presenting primarily with SVT is uncommon. In our case, it is difficult to postulate as to whether AMI precipitated the SVT or vice versa. It is possible that SVT in vulnerable patients can precipitate AMI by tachycardia induced shear stress resulting in plaque rupture and the hypotension promoting stasis and coagulation. The patient was advised treatment for coronary artery disease as well as electrophysiological study and radiofrequency ablation in case of tachycardia recurrences on medications.

## Figures and Tables

**Figure 1:**